# High-repetition-rate ultrafast electron diffraction with direct electron detection

**DOI:** 10.1063/4.0000256

**Published:** 2024-09-27

**Authors:** F. Rodrigues Diaz, M. Mero, K. Amini

**Affiliations:** Max-Born-Institut, Max-Born-Straße 2A, 12489 Berlin, Germany

## Abstract

Ultrafast electron diffraction (UED) instruments typically operate at kHz or lower repetition rates and rely on indirect detection of electrons. However, these experiments encounter limitations because they are required to use electron beams containing a relatively large number of electrons (≫100 electrons/pulse), leading to severe space-charge effects. Consequently, electron pulses with long durations and large transverse diameters are used to interrogate the sample. Here, we introduce a novel UED instrument operating at a high repetition rate and employing direct electron detection. We operate significantly below the severe space-charge regime by using electron beams containing 1–140 electrons per pulse at 30 kHz. We demonstrate the ability to detect time-resolved signals from thin film solid samples with a difference contrast signal, $\Delta I/I_{0}$, and an instrument response function as low as 10^−5^ and 184-fs (FWHM), respectively, without temporal compression. Overall, our findings underscore the importance of increasing the repetition rate of UED experiments and adopting a direct electron detection scheme, which will be particularly impactful for gas-phase UED. Our newly developed scheme enables more efficient and sensitive investigations of ultrafast dynamics in photoexcited samples using ultrashort electron beams.

## I. INTRODUCTION

Ultrafast electron diffraction (UED)^1–25^ is a powerful technique that tracks changes in the position of atoms within a material in real-time with picometre and femtosecond spatiotemporal resolution. UED is often employed in pump-probe configuration where an optical (“pump”) pulse photoexcites a sample away from its ground-state structure and another, time-delayed, electron (“probe”) pulse measures diffraction patterns of the excited sample. Pulses of electrons with high kinetic energies (in the keV or MeV) are easily attainable, providing an electron probe pulse with a (sub-)picometer de Broglie wavelength.^26–28^ The Fourier transform of the resulting diffraction pattern yields structural information with a spatial resolution of less than 10 pm.^18,21,23,28^ Measurement of diffraction patterns at various time delays between the two pulses allows the retrieval of a real-space “movie” of structural changes in the excited sample during a photochemical reaction.

**FIG. 1.**
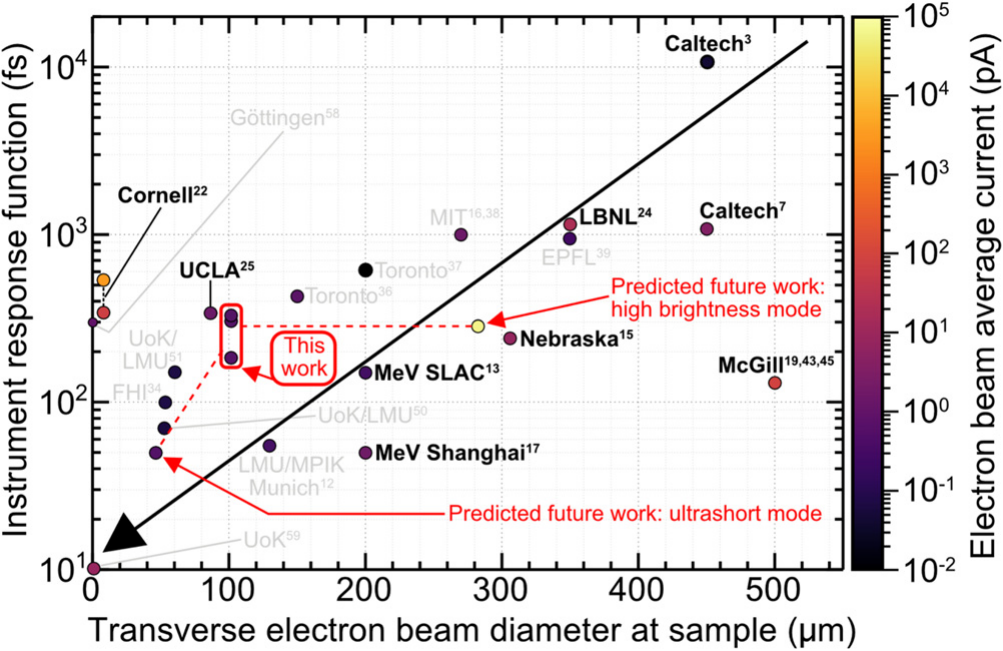
Evolution of instrument response function and transverse electron beam diameter at the sample position as a function of the average current of the electron beam. All values are given as full width at half maximum (FWHM) values. The diagonal black arrow indicates the evolution of UED toward shorter and smaller electron pulses. The electron flux of the electron beam is depicted by the z-scale colourmap representing the electron beam average current. Setups that are generally incompatible for gas-phase UED measurements are shown in gray text. Our work is highlighted by red text, arrows, and rectangles. The anticipated future capabilities of our setup are depicted by dashed lines, indicating the expected range of parameters.

In the early 1980s, Mourou, Williamson, and Li introduced the first picosecond variant of electron diffraction in transmission mode.^1,2^ The development of femtochemistry by Zewail^29,30^ enabled the use of femtosecond optical pulses to generate electron pulses.^3,7,31^ Despite this advancement, space-charge dispersion persisted in limiting the electron pulse duration to the picosecond regime.^3,7,31^ Following the ground-breaking contributions of Mourou^1,2^ and Zewail,^3,7,31^ extensive efforts have been dedicated to advancing the UED technique by numerous research groups and facilities. Figure 1 illustrates the impact of space-charge dispersion and the performance of different UED setups implementing diverse strategies. To reach a high spatiotemporal resolution, it is particularly important to generate an ultrashort electron pulse with the lowest pulse duration and transverse electron beam diameter. The electron pulse duration often dictates the total temporal resolution of UED measurements, referred to as the instrument response function (IRF). Reducing the transverse diameter of electron beams on-target minimizes the optical pump pulse diameter and, consequently, lowers the average power requirements of laser systems, which becomes particularly important when operating at high repetition rates. Furthermore, maintaining a high average beam current at the sample position is equally crucial to ensure the measurement of scattering signals with a high signal-to-noise ratio (SNR). This is particularly crucial in gas-phase UED studies, where electron scattering signals are approximately 100–1000 times weaker than those measured with solid thin films. Achieving the necessary high average beam currents in gas-phase UED is feasible by operating at high repetition rates. While cumulative heating effects may not be significant when studying simple metallic films, they become problematic when investigating more complex solid-state films that require a low base temperature.^32^ Our work aims to demonstrate the technical performance of our high-repetition-rate UED instrument using simple thin metallic films, in preparation for its use for future gas-phase UED studies, which are well-suited for operation at high repetition rates.

Realizing such optimal electron beam characteristics poses significant challenges owing to various constraints. These include limitations imposed by low repetition rates and severe space-charge effects, as well as issues associated with low electron scattering signals, non-optimal electron detection methodologies, and constraints related to electron beam flux and brightness. The relatively low repetition rate of laser systems driving UED setups necessitates the use of electron pulses with relatively high bunch charges (≫16-aC), leading to an increase in pulse duration from ∼150 fs to more than 1 ps in 1 m propagation due to severe space-charge dispersion for keV electrons. Siwick, Miller, and coworkers were the first to demonstrate sub-picosecond UED measurements.^33^ In these solid-state keV UED studies, space-charge effects were overcome by minimizing the sample-to-photocathode distance,^33^ often to less than 5 cm, and improving the initial electron beam characteristics.^34,35^ This optimization yielded an IRF as short as 100 fs and transverse beam diameters as small as less than 100 μm.^34^ However, instruments employing short sample-photocathode distances^16,34,36–39^ are generally incompatible with gas-phase UED experiments due to the higher operating pressures and the associated risk of voltage breakdown in the electron accelerator. So far, the generation of ultrashort electron pulses with durations of 100 fs or less over longer electron propagation distances has become feasible through several advancements. These include the utilization of relativistic MeV beams^9,13,14,17,20,21,40,41^ or the implementation of temporal compression schemes employing radio frequency (RF) fields generated by a microwave cavity^10,19,42–45^ or optical terahertz (THz) fields.^12,46–48^ Space-charge effects in 3-MeV electron beams are three orders-of-magnitude weaker than compared to <100-keV electrons at comparable bunch charge.^13^ This consequently enabled MeV-UED experiments with sub-150-fs time resolution.^9,13,14,17,20,21,40,41^ Notably, MeV-UED measurements with an IRF of sub-50 fs are now possible.^17^ However, access to accelerator-based MeV electron beams is confined to large-scale facilities, and achieving sub-10-fs time resolution with MeV setups necessitates precise synchronization of the RF accelerating field with the pump laser pulse.^20,25,43,49^ In keV UED instruments with a bunch charge much greater than 16 aC, the highest temporal resolution (FWHM) reported so far is approximately 100 fs in solid-state studies^19,50,51^ and 240 fs in gas-phase work.^15^ Furthermore, notable achievements include temporal compression of single-electron pulses to 28 fs (FWHM),^10^ the bunching of a 50-electron pulse into a train of 800-as single-electron pulses,^12^ and the use of the optical gating approach for attosecond electron diffraction.^52–54^

Additionally, ultrafast variants of transmission electron microscopes^53–59^ (UTEMs) have demonstrated electron pulses ranging from hundreds of femtoseconds^58^ to attoseconds^59,60^ duration, with nanometer-scale transverse focused beam diameters^24,58,61^ reaching down to 9 Å.^58^ Performing gas-phase measurements using UTEMs is technically challenging. Therefore, in this work, we will focus solely on UED setups capable of performing gas-phase UED measurements. It is important to note that in electron beam physics, the 6D bunch brightness^57^ is generally a better figure of merit than the electron flux. This is particularly crucial for solid thin film samples, which suffer from thermal effects at relatively high repetition rates and require small (few μm or less) electron beam diameters, along with tailored sample thin films using nm-scale gold masks.^62^ However, for gas-phase measurements, the smallest transverse beam size does not necessarily need to be on the few μm or smaller scale. This is because the length of a typical gas jet is at least 100–200 μm (Refs. 13, 44, and 63) and even extend to the millimeter scale in the case of MeV beams using flow cells.^63^ To achieve a suitable signal-to-noise ratio in gas-phase UED, a minimum transverse beam diameter of at least approximately 50–100 μm is ideally desired. Such a transverse beam diameter will still be sufficient to alleviate the average power ( ≥200 W) requirements for performing UED measurements with a low bunch charge (<16-aC)^12,50,51^ at higher repetition rates ( ≥100 kHz) than those currently employed.

Electron detection with a minimal noise contribution to the SNR is another crucial factor. High-energy electron beams are often detected using a phosphor scintillator screen, which emits a few hundred photons for each detected electron.^64^ The emitted photons are subsequently measured by either a fiber- or lens-coupled photon-sensitive imaging sensor. These sensors, which include charge-coupled device (CCD),^65^ electron-multiplying CCD (EMCCD),^13^ or complementary metal oxide semiconductor (CMOS)^64^ technology, are susceptible to saturation effects from bright signals. For example, saturation from the unscattered beam is typically mitigated by employing a beam blocker positioned in-front of the phosphor screen. However, this approach results in the loss of valuable information on fluctuations in the electron beam's intensity and pointing, which are crucial for correcting these fluctuations. Furthermore, these charge-integrating analog detectors also suffer from relatively larger gain, integration, and readout noise, limiting the SNR that can be achieved.^64^ Additionally, these indirect electron detection schemes often exhibit a limited dynamic range, particularly at high momentum transfers. It is worth noting that microchannel plates (MCPs), positioned in-front of a phosphor scintillator screen,^65^ have also been used; however, their detection efficiency of high-energy electron beams (<15%) is not ideal.^66^

In this work, we employ a novel, high-repetition-rate UED (HiRepUED) instrument operating at 30 kHz, utilizing direct electron detection. Our approach involves the use of temporally uncompressed electron pulses containing between 1 and 140 electrons, corresponding to a bunch charge of 0.16–24 aC. This bunch charge is significantly lower than that employed in existing (sub-)kHz UED setups (2–20 fC), leading to significantly weaker space-charge forces and lower emittances in keV beams, allowing us to operate in the no-to-moderate space-charge regime. We find that temporally uncompressed electron pulses as short as 174 fs are achievable, resulting in an IRF of ∼184 fs. This IRF is better than that reported for the keV UED instrument utilized by Centurion and coworkers (240 fs) but using an RF-compressed electron scheme.^15^ Furthermore, we demonstrate the measurement of time-resolved signals with improved SNR using direct electron detection as compared to indirect detection schemes. As a future outlook, we also discuss the future potential of our instrument with RF temporal compression, and give a brief discussion of its anticipated performance when operating in the ultrashort and ultrabright modes (see dashed lines in Fig. 1).

This paper is structured as follows. Details of the experimental setup and simulations are given in Secs. II and III, respectively. Results obtained from measurements and simulations are discussed in Sec. IV, and a summary conclusion is given in Sec. V.

## II. EXPERIMENTAL

### A. Optical setup

**FIG. 2.**
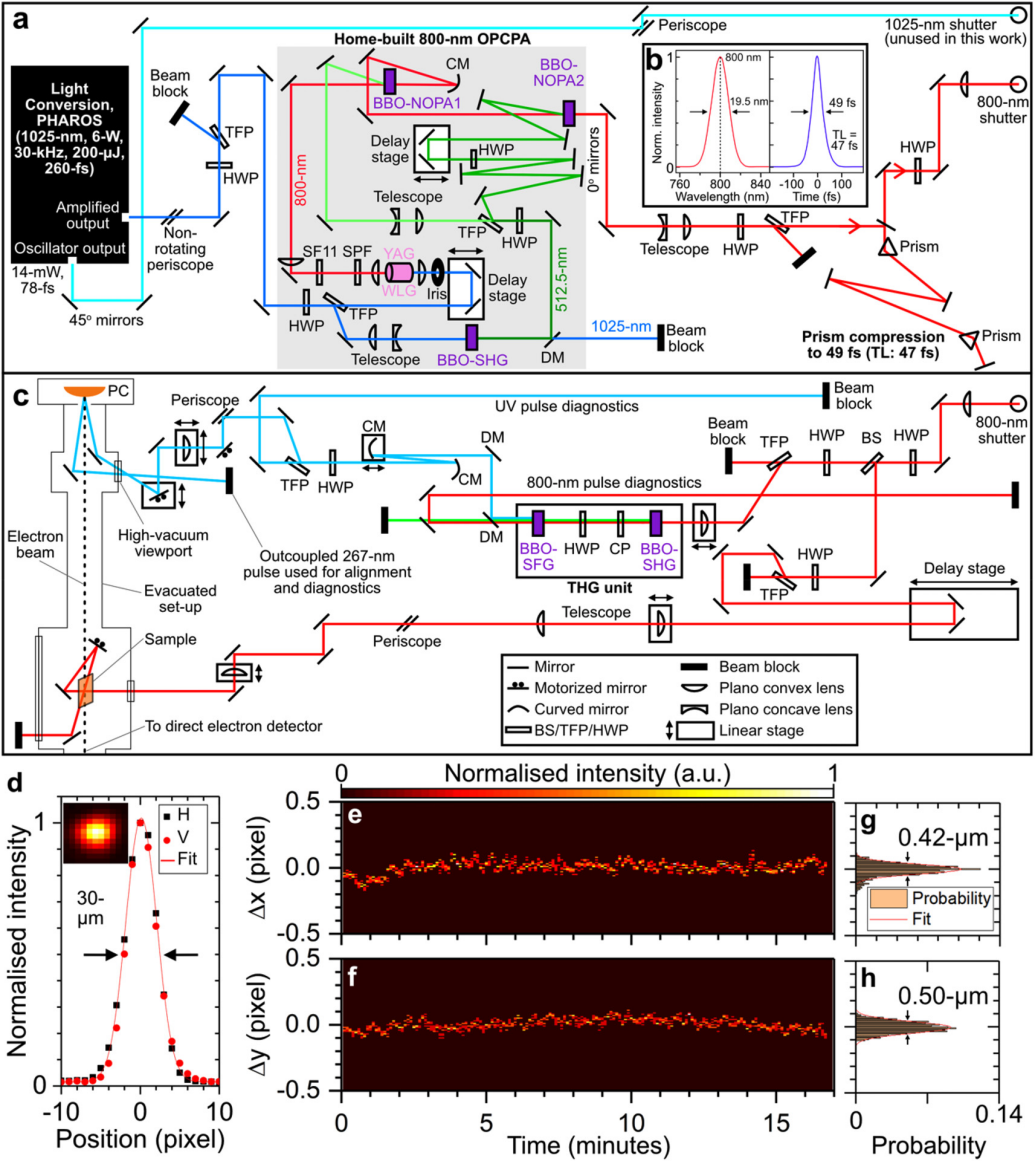
(a) Schematic of a 30-kHz, home-built optical parametric chirped pulse amplification (OPCPA) system that employs a 6-W PHAROS laser system as the supercontinuum-seed and pump laser, and subsequent prism compression. (b) Wavelength and temporal profile of OPCPA output reconstructed from frequency-resolved optical gating (FROG) measurements. The central wavelength, $\lambda_{c}$, and transform limit (TL) of the pulse are indicated. (c) Schematic of optical setup for generating electron probe pulses using ultraviolet (UV) light at the photocathode (PC), and sample excitation using 800-nm optical pump pulses. (d) UV beam profile. Cuts along the horizontal (H) and vertical (V) axes are shown with a Gaussian fit. Inset: UV beam detector image. (e) and (f) UV beam pointing measurement. Changes in the (b) horizontal ( Δx) and (c) vertical ( Δy) position of the UV beam are shown. (g) and (h) Histogram distribution of the corresponding data shown in panel (b) and (c). Gaussian fits were applied to the histogram distributions.

Figure 2 shows a schematic of the optical setup employed in this study. Here, the amplified output of a 30-kHz, 200-μJ laser system (Light Conversion PHAROS, 1025-nm, 6-W, 260-fs) was split into two beams using a half waveplate (HWP) and a thin film polarizer (TFP) positioned at Brewster's angle [see Fig. 2(a)]. The p-polarized transmitted pulse was used as a supercontinuum seed pulse for white light supercontinuum generation (WLG) in the home-built optical parametric chirped-pulsed amplification (OPCPA) setup. The s-polarized reflection was frequency converted to 512.5 nm (220-fs FWHM, 100-μJ) and was used as the pump beam of two non-collinear optical parametric amplification (NOPA) stages. The footprint of the OPCPA was 60 × 45 cm^2^. The signal output of the OPCPA ( $\lambda_{c}=$ 800 nm, ∼17 μJ, ∼190 fs) was temporally compressed to 49 fs using prism compression [see Fig. 2(b)] close to its Fourier transform limit (TL) of 47 fs [19.5 nm FWHM bandwidth, see Fig. 2(b)]. The compressed near-infrared (NIR) 800-nm pulse was split into two beams using a 20:80 beam splitter [see Fig. 2(c)] where one beam was used for electron generation, while the second beam was used for sample photoexcitation.

In the electron generation (probe) beam, the NIR pulse (3-μJ) was frequency converted to 267 nm (20 nJ, 90 fs, TL: 42 fs) using a compact third harmonic generation unit (Eksma Optics, FemtoKit) composed of two BBO crystals, a HWP and a calcite plate (CP). Two harmonic separating dichroic mirrors (DMs) separated the 267-nm ultraviolet (UV) pulse from the residual 400-nm and 800-nm pulses. The UV pulse was subsequently beam expanded using two curved mirrors (CMs) by a magnification factor of two, producing a collimated UV beam (∼5-mm FWHM diameter). The UV pulse was then power attenuated using a HWP and TFP positioned at Brewster's angle. The p-polarized transmitted pulse was used for UV pulse diagnostics and alignment purpose. The s-polarized reflection was directed to the UED instrument, where it was focused to a 30-μm FWHM diameter spot on the photocathode [see beam profile in Fig. 2(d)]. The ultraviolet (UV) pulse duration at the photocathode was estimated to be 90 fs based on the group dispersion delay (GDD; ∼1200 fs^2^) expected from the UV optics employed in our optical setup [see Fig. 2(b)]. The pointing stability of the UV pulse at the focus was measured to be ∼0.50 μm using a UV-sensitive beam profiling camera (PCO.edge 4.2 bi UV) over a ∼15-min period, as shown in Figs. 2(e)–2(h). Additionally, an intensity jitter of 1% (FWHM) was observed during this period. As the UV pulse is generated through non-linear conversion using an 800-nm pulse, the above-mentioned jitter values of the UV pulse can be taken as the maximum corresponding values for the 800-nm pulse. Notably, our optical setup does not employ a beam pointing stabilization system.

In the sample photoexcitation (pump) beam, the NIR pulse energy (12 μJ) was attenuated using a HWP and TFP at Brewster's angle, and subsequently reflected by a retroreflector mounted on a linear delay stage (Physik Instrumente M-531.2S1, 30-cm travel range). The NIR pulse was beam expanded and subsequently focused to a ∼180-μm-diameter spot at the sample position using a plano-convex uncoated lens ( f = +600 mm). The relative angle of the optical pump pulse to the electron axis was ∼20 °. The mirror after the NIR lens and the last mirror before the sample are piezo-mounted to obtain the optimal spatial overlap with the electron beam at the sample position.

### B. Experimental setup

**FIG. 3.**
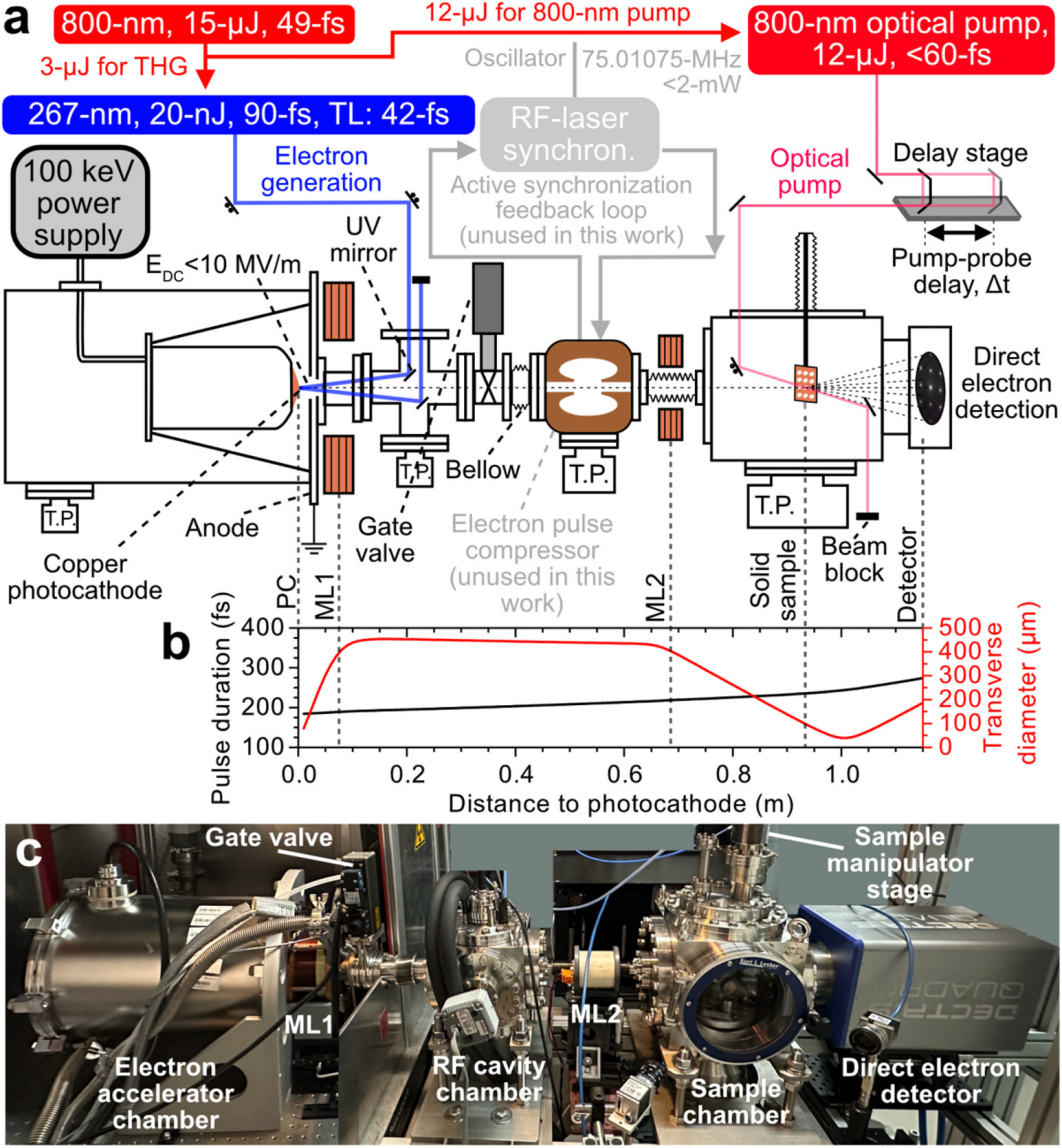
(a) Schematic of the 30 kHz, 95 keV HiRep-UED instrument, with each component labeled. See main text for more details. (b) General Particle Tracer (GPT) simulation of the electron pulse duration (blue distribution) and transverse diameter (red distribution) with the instrument operating in the temporally uncompressed mode using an electron beam containing 55 electrons. All values are given in FWHM. (c) Image of the instrument in the laboratory.

Figure 3 shows a schematic of the HiRep-UED instrument employed in this study. The UV pulse was focused to a 30-μm FWHM diameter spot [see Fig. 2(d)] on a high-purity oxygen-free copper photocathode (>99.99%).^67^ Since the photocathode was held at a high voltage of 95 keV with a high voltage power supply (Matsusada AU-100N1.5-L), the effective work function of copper was reduced due to the Schottky effect (∼4.3-eV)^67^ such that the photon energy of the UV pulse (4.65 eV) was sufficiently high to generate electrons via photoemission. A grounded anode flange positioned ∼11 mm away from the photocathode generated a constant direct current (DC) field with a field strength of E_DC_ < 10 MV/m, sufficient to accelerate the electron beam to 95 keV. The electron beam passed through a 10-mm aperture and was transversely collimated and focused by two solenoid magnetic lenses (MLs) labeled ML1 and ML2, respectively. The collimating solenoid magnetic lens ML1 (Doctor X Works) was placed directly in-front of the anode flange located 40 mm away from the photocathode. ML1 consisted of rectangular (2.5 × 1.5 mm^2^) copper wire wound into a coil with 351 turns, an inner and outer diameter of 42.5 and 68.5 mm, respectively, and a length of 60 mm. The condensing solenoid magnetic lens ML2 (Doctor X Works) was placed ∼670 mm away from the photocathode. ML2 comprised of circular (1-mm-diameter) copper wire wound with 650 turns forming a 60-mm length coil with an inner and outer radius of 20 mm and 37.5 mm, respectively. The center hole is 35 mm in diameter. A maximum on-axis magnetic field flux density of 40 mT (17.5-mT) at 12 A (1.75 A) for ML1 (ML2) can be generated. The focused electron beam scattered against thin-film solid-state samples held by a 0.5-mm-thick copper block capable of holding up to nine samples with 3 mm diameter. The sample holder was mounted onto a manual four-axis (x, y, z, $\theta_{\text{yaw}})$ ultrahigh vacuum mechanical translator stage (VAb Vakuum-Anlagenbau). An RF microwave cavity (Doctor X Works) for temporal electron compression was installed together with an active synchronization system that corrects the RF-laser timing jitter based on Ref. 43. However, both are unused in this work as it is beyond the scope of the current work and is subject of a separate publication.

The scattered and unscattered electrons are measured with a direct electron detector (DECTRIS QUADRO)^68,69^ composed of 512 × 512 pixels of 75 × 75 μm^2^ size, giving a total active area of 38.4 × 38.4 mm^2^. Each pixel was composed of a 450-μm thin silicon wafer. This combination ensures a detection efficiency of more than 90% for 95-keV electrons (i.e., single-electron detection). Each pixel can detect up to four electrons per pulse (see Sec. IV B), while possessing a 32-bit dynamic detection range (sum of two 16-bit electron counters), capable of counting up to 4.2 × 10^9^ hits in a given exposure time. The detector's maximum count rate was determined as 10^7^ electrons/second/pixel under continuous illumination.^68^ Loss of sensitivity was observed with the detection of an unfocussed electron beam containing more than 10^4^ electrons at 30 kHz, with the beam covering the full sensor dimensions. In addition to the electron detector, a home-built Faraday cup (not shown in Fig. 3; 2 mm open aperture diameter, 20 mm length) positioned in-front of the detector was used to measure the average current of the electron beam containing more than 10^4^ electrons. Practical acquisition times for time-resolved measurements at 30 kHz spanned from between 20 and 90 min for data shown in this work. Typical instrument parameters of the HiRep-UED setup are summarized in Table I. Furthermore, details of the HiRep-UED instrument are provided in Sec. B of the supplementary material.^103^

**TABLE I.** Typical machine parameters for the HiRep-UED setup in this work.

Parameters	Values
Repetition rate	30 kHz
Vacuum in the following chambers	
Electron accelerator	<7 × 10^−7^ mbar
RF cavity	<3 × 10^−8^ mbar
Sample chamber	<3 × 10^−8^ mbar
Electron beam kinetic energy	95 keV
Electron beam charge	0.16–22 aC (1–140 electrons/pulse); overall range is 0.16 aC–1.6 pC (i.e., 1 to 10^6^ electrons/pulse)
Electron flux	3.0 × 10^4^ to 4.2 × 10^6^ electrons/s (maximum of 3.0 × 10^10^ electrons/s)
At the photocathode position	
UV pulse size (FWHM)	30 μm
UV pulse duration (FWHM)	90 fs (TL: 42 fs)
UV pulse energy	<20 pJ (overall range of 1 pJ–20 nJ)
At the sample position	
Electron beam charge (after passing through aperture)	<0.16–6.8 aC (<1–42 electrons/pulse)
Electron bunch length (FWHM)	174–322 fs (uncompressed) (<50 fs compressed predicted by GPT)
Electron beam size (FWHM)	∼100 μm (200 μm)
Electron beam transverse pointing jitter (FWHM)	≤ 28 μm
Transverse coherence length (RMS)	3.8 nm
Transverse emittance (RMS)	3.8 nm rad
Longitudinal emittance (RMS)	103–177 fm rad
Pump laser spot size (FWHM)	∼180 μm
Pump laser duration (FWHM)	<60 fs (TL: 47 fs)
At the detector position	
Electron beam size (FWHM)	∼450 μm (280 μm)
Reciprocal-space resolution	0.063 Å^−1^
Spatial resolution	3.8 pm

## III. SIMULATIONS

### A. General particle tracer simulations

General particle tracer (GPT)^70–72^ simulations of the UED instrument were performed using an electron beam with a kinetic energy of 95 keV and an excess thermal energy of 0.5 eV. The electron beam was generated by a UV pulse with a FWHM diameter and duration of 30 μm and 90 fs, respectively, without the use of the RF cavity (i.e., temporally uncompressed mode). For details about the influence of the UV pulse duration on the temporally uncompressed electron pulse, the reader is referred to Sec. C and Fig. S2 of the supplementary material.^103^ For comparison, simulations were also performed with RF-compressed electron beams (i.e., temporally compressed mode), using a UV pulse duration of 60 fs. Field maps of the electron accelerator and solenoid magnetic lenses, generated from finite element method (FEM) simulations, were used. For each simulation, we used 10 000 macroparticles with the mesh method.

## IV. RESULTS AND DISCUSSION

### A. Electron beam characterization

#### 1. Electron flux

**FIG. 4.**
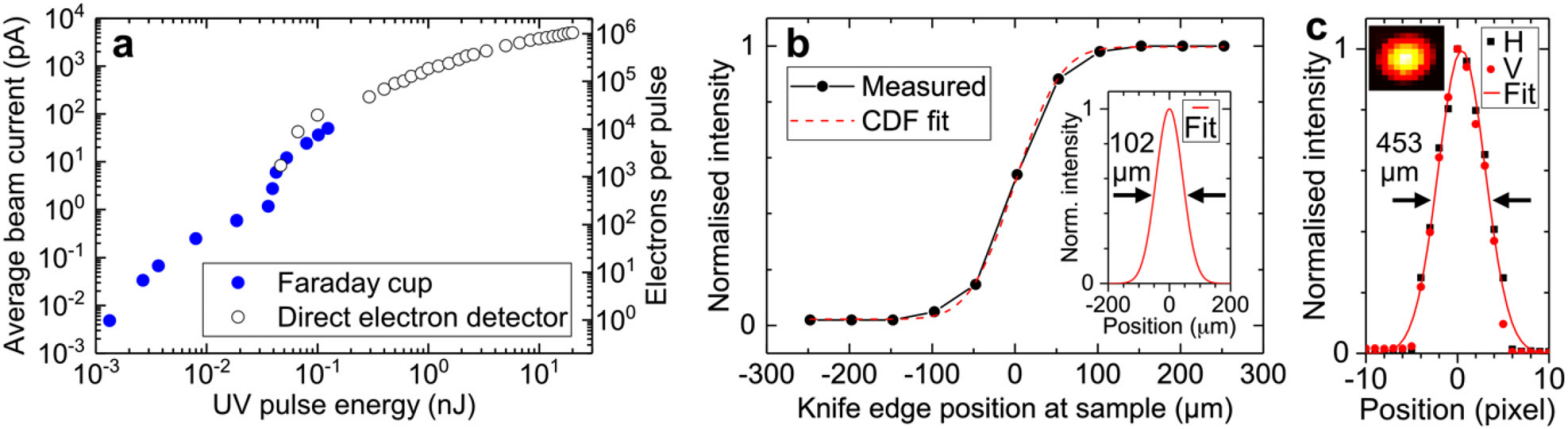
(a) Average beam current and electrons per pulse as a function of UV pulse energy measured with a Faraday cup and direct electron detector, respectively. (b) Knife-edge scan of electron beam at the sample position. A Gaussian cumulative distribution function (CDF) was fitted to the measured data. Inset: retrieved Gaussian distribution from CDF fit in panel (b). The FWHM electron beam diameter is indicated by black arrows. (c) Electron beam profile at the detector position obtained from the same data as in panel (b). Inset: electron detector image.

Our UED setup is capable of generating an electron pulse containing between one and >10^6^ electrons at 30 kHz with a UV pulse energy between ∼1 pJ and 20 nJ, as shown in Fig. 4(a). Operating in high-brightness mode, our setup achieves an unprecedented electron flux exceeding 10^10^ electrons/s, surpassing existing keV-scale UED systems by more than an order of magnitude.^15,19,22,25,44^ Compared to the brightest MeV-scale UED setup,^13^ our system has a 100-fold higher electron flux. Considering the factor of four higher elastic electron scattering cross section for 95-keV electrons compared to 3-MeV electrons, calculated with ELSEPA,^73^ the maximum electron scattering signal from our setup is approximately 400 times greater than the current state-of-the-art in MeV-UED.^13^ Even in anticipation of upgrades to MeV-UED facilities aiming for a 1-kHz repetition rate, the difference in signal level between our setup and MeV-UED remains appreciably high (factor of ∼150).

#### 2. Electron transverse profile at sample and detector

We performed measurements to determine the full width at half-maximum (FWHM) diameter of an electron beam containing 100 electrons. Using specific combinations of ML1 and ML2 currents, we adjusted the transverse focus of the beam to be positioned either close to the sample or the detector. Knife-edge scans were employed to measure the electron beam size at the sample position. The electron beam size at the detector was measured directly on the direct electron detector. In our typical operating configuration, with the transverse focus at the sample, we measured beam diameters of 102 and 453 μm at the sample and the detector, respectively [see Figs. 4(b) and 4(c)]. With a different combination of ML1 and ML2 currents, we were able to reduce the electron beam diameter to approximately 280 μm at the detector, albeit with a larger 200 μm diameter at the sample. Figure 3(b) shows corresponding General Particle Tracer (GPT) simulations of the transverse diameter of an electron pulse containing 55 electrons in our UED instrument (i.e., 8.8 aC; see red line). The initially divergent electron beam is collimated by ML1 to a diameter of 450 μm (FWHM) at the position of the RF cavity (∼0.5 m from photocathode). The collimated beam is subsequently focused by ML2 to 100 μm (FWHM) close to the sample position (∼0.95 m from photocathode). A good agreement between the measured (102 μm) and simulated (100 μm) transverse beam diameter at the sample position is observed. The GPT simulations further predict an RMS transverse and longitudinal emittance at the sample of 5.6 nm rad and 103 fm rad (10.5 nm rad and 11.4 pm rad), respectively, for a bunch charge of 8.8 aC (16-fC). Interestingly, the measured RMS transverse emittance is determined as 3.8 nm rad (see Sec. D and Fig. S3 of the supplementary material^103^) using knife-edge scans of the electron beam at different currents of ML1. The discrepancy between measured (3.8 nm rad) and simulated (5.6 nm rad) transverse emittance is most likely due to an overestimation of the mean transverse energy (MTE; ∼0.5 eV) in GPT simulations often determined within the severe space-charge regime. In reality, the MTE is lower when operating the instrument in the no-to-low space-charge regime (i.e., <0.5 eV).

#### 3. Reciprocal-space resolution and transverse coherence length

**FIG. 5.**
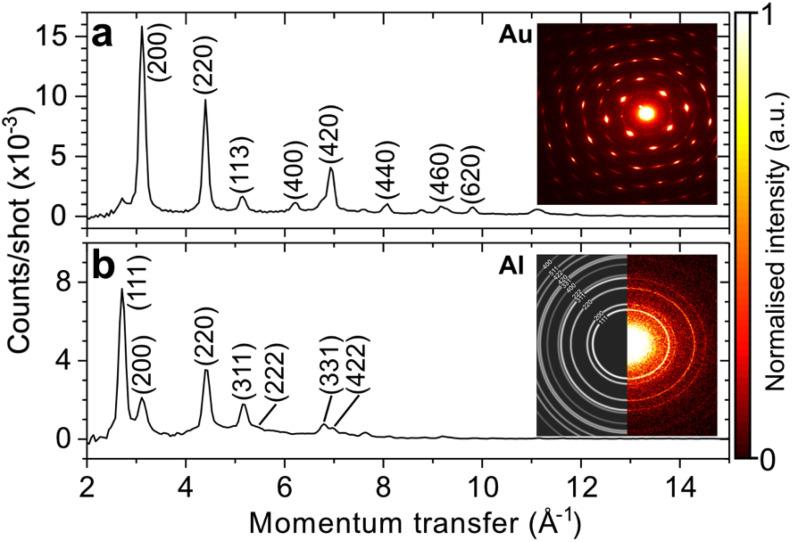
Electron diffraction patterns of (a) 11-nm monocrystalline gold (Au) and (b) 31-nm polycrystalline aluminum (Al) measured using a 95-keV electron pulse containing ∼100 electrons at 30-kHz with a direct electron detector. Both data were measured without apertures. Insets: measured detector images, with simulated data shown for aluminum.

The reciprocal-space resolution and transverse coherence length of our instrument were characterized experimentally by measuring the electron diffraction pattern from a monocrystalline gold film (Plano GmbH, 11 nm) and a polycrystalline aluminum film (Plano GmbH, 31-nm) using a 95-keV electron pulse containing ∼100 electrons on-target. These reference samples are often used to characterize both gas-phase and solid-state UED instruments. Figure 5(a) shows distinct Bragg diffraction spots from the monocrystalline film. Our instrument was further characterized for isotropic electron diffraction signals, which are typically measured in gas-phase UED, by measuring the diffraction pattern generated from the polycrystalline film, as shown in Fig. 5(b). From the FWHM of the first-order diffraction peaks, we obtain a reciprocal space of 0.063 Å^−1^. An excellent agreement is observed between measured and simulated data, the latter of which is calculated using a powder electron diffraction simulation software (CrystalMaker^®^).^74^ Furthermore, the RMS transverse coherence length, *ε*_co_, of our UED instrument was experimentally characterized using the method described in Ref. 11 that takes into account the reciprocal-space RMS widths of and distance between two closely lying Bragg electron diffraction signals. Here, we used the electron diffraction signals corresponding to the (hkl)=(220) and (420) Bragg peaks in gold monocrystalline film and the (hkl)=(220) and (311) peaks in polycrystalline aluminum. We obtain a *ε*_co_ value of 3.8 nm from both data.

#### 4. Electron pulse pointing and intensity jitter

**FIG. 6.**
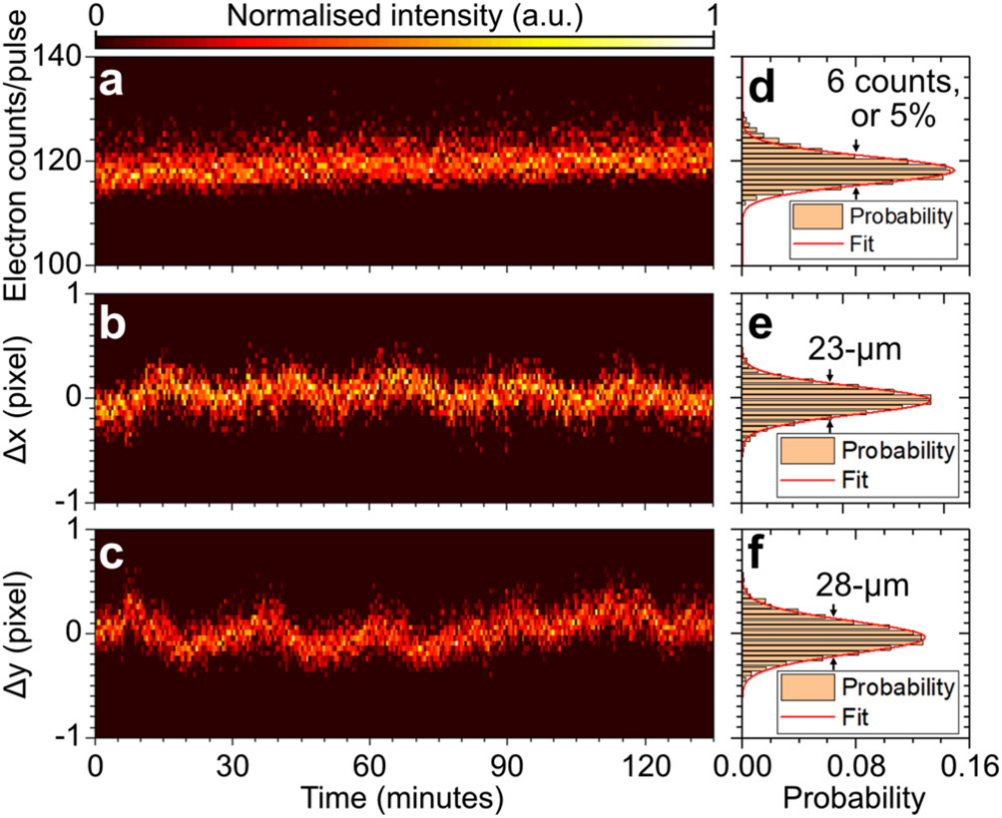
Two-dimensional plot of the (a) number of electron counts per pulse, and changes in the (b) x-center position ( Δx), and (c) y-center position ( Δy) as a function of time. A total of ∼8100 images were acquired, with each image capturing the electron beam, which had an average of ∼110 electrons, over an exposure time of 1 s. (d)–(f) Histogram plots of the data corresponding to panels (a)–(c) with a Gaussian fit applied. All values are given in FWHM.

Figure 6 shows the long-term pointing and intensity drift of the electron beam measured with a direct electron detector over a two hour period. The beam pointing and intensity fluctuations of the electron beam were characterized experimentally [see Figs. 6(d) and 6(f)]. A Gaussian fit to the histogram distribution of electron intensity in Fig. 6(d) shows that the electron beam has a 5% (FWHM) intensity fluctuation, while a horizontal and vertical pointing jitter of 23 and 28 μm, respectively, are observed [see Figs. 6(e) and 6(f)].

#### 5. Comparison to other UED instruments

The transverse diameter and reciprocal-space resolution of the electron beam in our instrument is a factor of two-to-three smaller than other instruments employing bunch charges exceeding 8.8-aC.^13,15,19,44^ This reduction is attributed to a lower transverse emittance enabled by the use of an electron beam with a reduced bunch charge (8.8 aC). The measured transverse emittance at 8.8 aC (3.8 nm rad) is nearly a factor of ten smaller than that reported for most other instruments typically operating at 16 fC (10 nm rad).^22,23,75^ Moreover, the larger RMS transverse coherence length, *ε*_co_, of our UED instrument (3.8 nm) compared to the state-of-the-art (∼3 nm)^11,13,76^ has important implications for coherently imaging larger lattice unit cell and molecular structures. For example, for solid-state samples, a larger *ε*_co_ would mean that the lattice unit cell could be probed an additional number of times, improving the signal-to-noise ratio of the measured electron diffraction signal. Notably, sub-nm rad emittance, nm-scale transverse electron beam diameters, and coherence lengths of 10 nm or higher have been demonstrated with the next generation of photocathode materials.^22,24,58,61,77^ Furthermore, the pointing stability of our instrument ( ≤ 28-μm) is similar to that reported for the MeV-UED instrument at SLAC (33-μm).^13^

### B. Direct electron detection

#### 1. Saturation effects in a direct electron detector

**FIG. 7.**
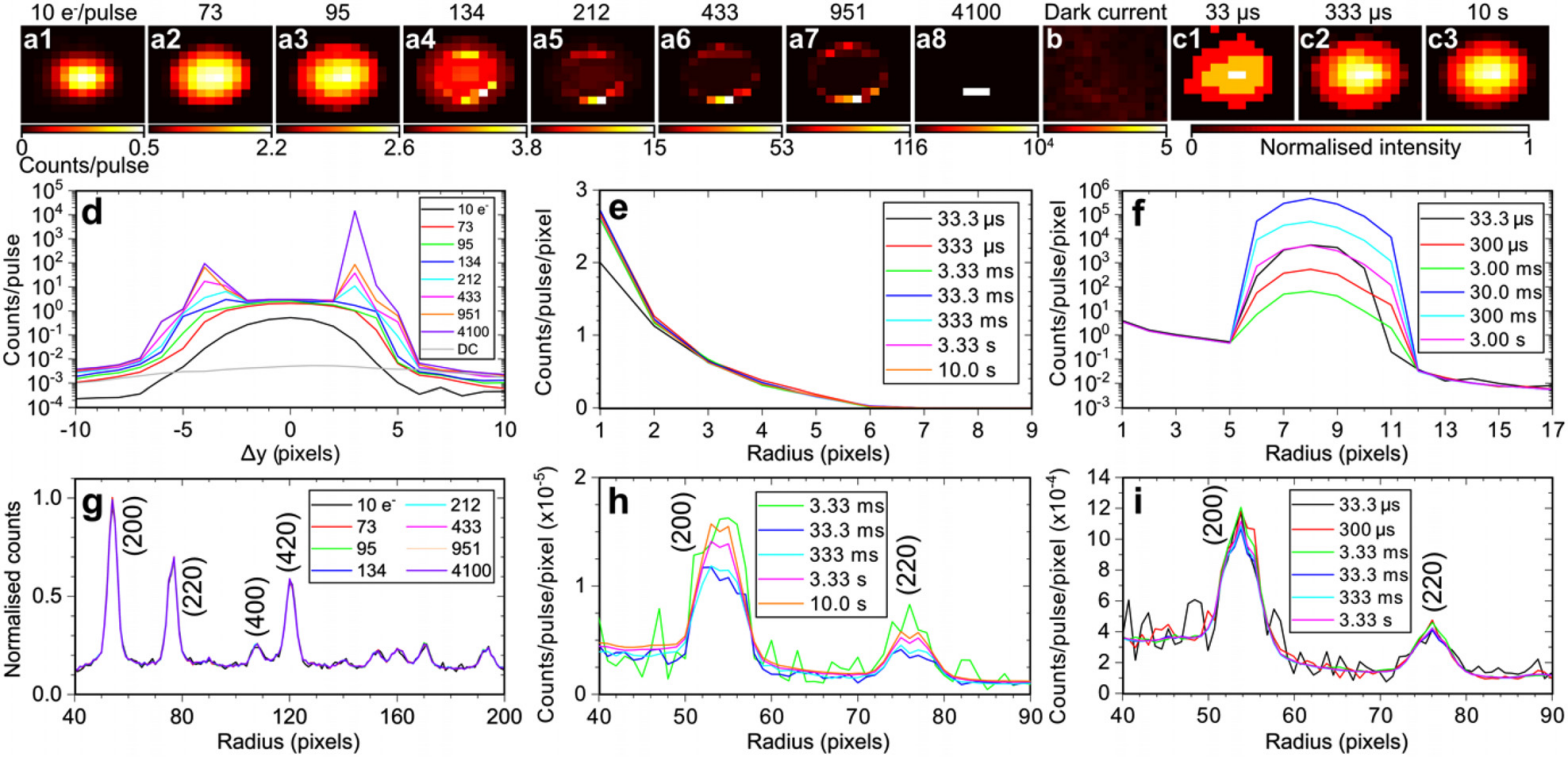
Saturation effects in DECTRIS QUADRO direct electron detector. (a1)–(a8) Detector images measured with a different number of electrons per pulse as indicated at the top of images 1–8. Exposure time of 0.1 s was used. (b) Detector image of dark current contribution with a colourmap scaling that was multiplied by a factor of 10 relative to that of image a1. Exposure time of 0.1 s was used. (c1)–(c3) Detector images of an electron beam containing 10^2^ electrons measured with different exposure times. (d) Vertical beam profile of the primary unscattered electron beam after interaction with an 11-nm monocrystalline gold sample using an electron pulse containing different numbers of electrons. (e) and (f) Radial distribution of electron beam measured with different exposure times using an electron beam containing (d) 10^2^ electrons and (e) 10^4^ electrons. (g)–(i) Radial distributions of Bragg diffraction peaks from the sample corresponding to data from panels (d)–(f). Panel (g) was normalized to the sum of the total intensity corresponding to a radius of 30–200 pixels for a like-for-like comparison of data measured with different number of electrons. Panels (a), (b), (d), and (g) were averaged over 100 images, while all other panels were measured with a single image.

Figure 7(a) shows detector images of the unscattered electron beam containing a varied number of electrons, from 10 electrons/pulse to more than 4100 electrons/pulse, recorded after passing through a monocrystalline 11-nm gold thin film sample. Saturation effects become apparent using an electron pulse containing 134 electrons [see Fig. 7(a4)]. For example, the vertical profile of the electron beam [see Fig. 7(d)] is modified from a Gaussian distribution below saturation to a bimodal distribution when the central pixels become saturated by an electron beam containing 134 electrons or more. Furthermore, in the case of more than 4100 electrons/pulse [see Fig. 7(a8)], significant saturation effects occur; the central pixels detecting the central portion of the electron beam deviate from a normal counting detector behavior. We note nevertheless that when the central pixels of the detector are saturated by the unscattered electron beam, the scattered signal observed at larger radii on the detector is not affected, as shown in Fig. 7(i). This demonstrates that every pixel operates as an independent electron counter. This is in contrast to the typically-employed CCD,^65^ EMCCD,^13^ and CMOS^64^ detectors.

**FIG. 8.**
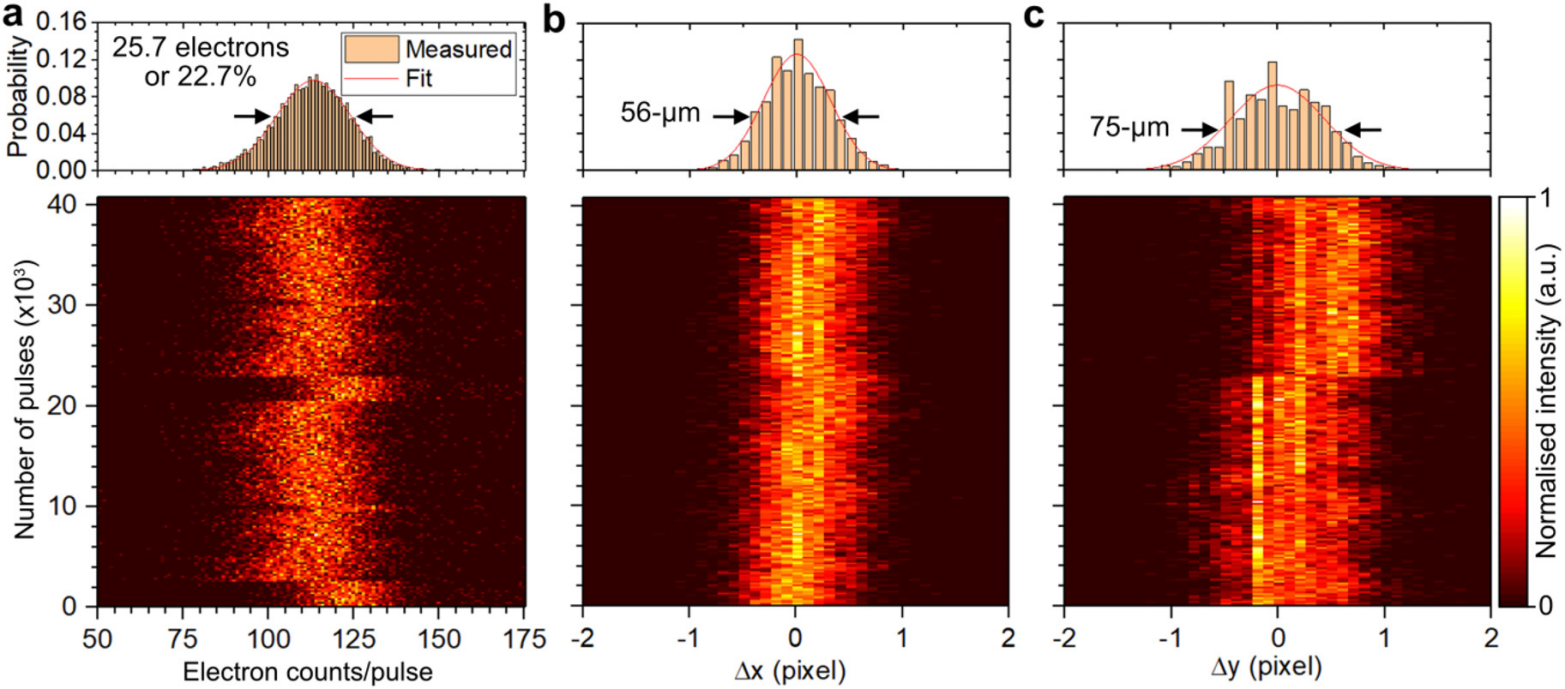
Shot-to-shot analysis. (a) Two-dimensional plot of the number of electrons per shot as a function of number of pulses. An electron beam containing an average of 113 electrons was measured with an exposure time of 33.3 μs. A total of 40 000 images were recorded. A histogram plot of the associated data is shown at the top of the panel with a Gaussian fit applied. Same as in panel (a) but for changes in the x (b) and y (c) center positions of the electron beam.

Furthermore, with an exposure time of 33 μs (i.e., 1 shot), the radial distribution of an electron beam containing 100 electrons is measured with a high SNR, while an exposure time of at least 3.33 ms is required to measure the first-order (200) and (220) Bragg diffraction peaks of monocrystalline gold with an SNR of four [see Fig. 7(h)]. Measurement of electron signals significantly above the pixel saturation threshold [see Fig. 7(f)] reveals that the corresponding pixels behave like a paralyzed counting detector,^78^ with signal decreasing as exposure time increases from 33.3 μs to 3.0 ms. Moreover, a sudden intensity surge is observed at an exposure time of 30.0 ms, followed by a decline in signal at longer exposure times. Given the presence of two 16-bit counters in each pixel, this observed saturation behavior is attributed to the initial paralysis of the first 16-bit counter, followed by the subsequent paralysis of the second 16-bit counter. Notably, in this regime, an exposure time of 33.3 μs (i.e., a single shot) is sufficient to measure the first-order diffraction peaks with an SNR of four [see Fig. 7(i)], demonstrating the high sensitivity of this detector.

Specifically, each pixel of this detector utilizes a retrigger mechanism, which counts the length in time that the amplitude of the measured signal is above a predefined value corresponding to a threshold energy (in our case 12 keV). This length in time is measured as multiples of a programmable time, called the retrigger time (i.e., the time that the signal from one electron is above threshold). The amplitude of the signal is approximately proportional to the energy deposited by the 95-keV electron in that pixel. Therefore, when multiple electrons impinge on the same pixel, this generates a signal with an amplitude that is the sum of the energy deposited by each electron. At every retrigger time that the signal is still above threshold, a count is added to the electron counter. Thus, longer time-over-threshold durations enable the QUADRO to distinguish multiple hits from a single hit.^68,79^ Under continuous illumination, a limit of 10^7^ electrons/second/pixel with a retrigger time of 10 ns was established.^68^ In our pulsed operation, conducted below saturation, our analysis from Figs. 7(d) and 7(e) indicates that approximately three electrons per pulse can be detected by a single pixel (i.e., $9\times 10^{4}$ electrons/second/pixel, and a retrigger time of 825 ns). Thus, the QUADRO's retrigger feature enables the counting of up to three electrons arriving within the electron pulse duration (<350-fs) in a single pixel at 30 kHz.

#### 2. Shot-to-shot jitters and their correction

We have characterized the electron beam's intensity and pointing fluctuations on a shot-to-shot basis. To demonstrate the capability to correct for such fluctuations, we performed measurements immediately after applying current to the two magnetic solenoid lenses, without allowing time for thermalization, to ensure maximum fluctuation in the electron beam. Employing a similar histogram analysis as that presented earlier, we find that an electron beam containing 113 electrons exhibited a FWHM intensity jitter of 25.7 electrons or 22.7%, as shown in Fig. 8(a). This is approximately equivalent to the shot noise limit assuming Poisson statistics (∼22.2%).^64^ Furthermore, changes in the electron beam's center position, Δx and Δy, were 56 and 75 μm, respectively, as shown in Figs. 8(b) and 8(c).

**FIG. 9.**
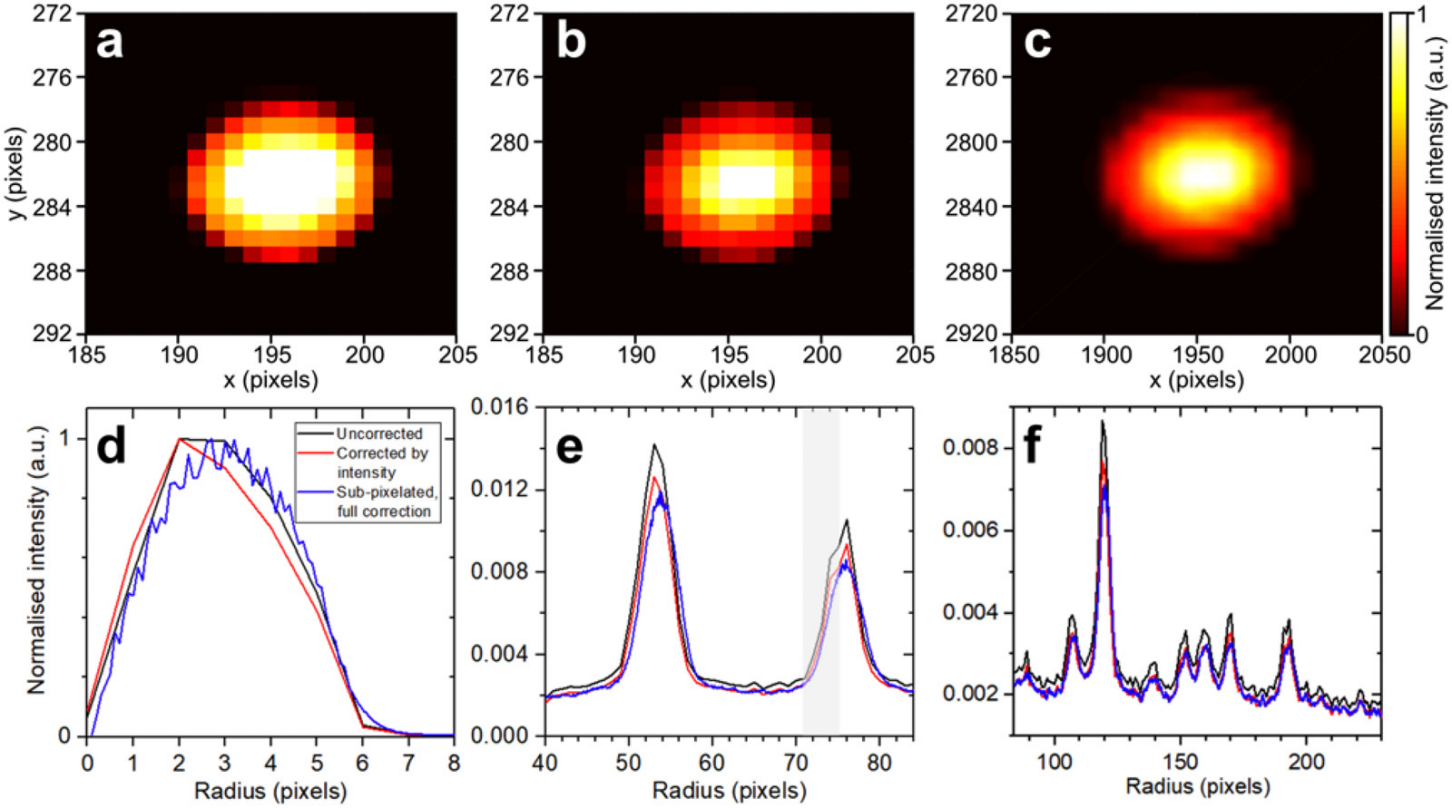
Shot-to-shot correction of electron beam pointing and intensity jitter with sub-pixel accuracy. Summed detector image of electron beam (a) before any correction, (b) after intensity jitter correction, and (c) after intensity and pointing jitter correction with sub-pixelation of factor 10. The images shown were summed over ∼40 000 shots corresponding to the data shown in Fig. 8. (d) Radial distribution of primary unscattered electron beam before and after correction. (e) and (f) Radial distribution of Bragg diffraction peaks from 11-nm monocrystalline gold before and after correction.

An algorithm was developed that implements multiple corrections on a shot-to-shot basis, applicable to data measured with any direct electron detector. Initially, each element within the 512 × 512 pixel matrix of every image was normalized by the total number of electrons present in the unscattered beam. The resulting sum detector image before and after intensity correction is shown in Figs. 9(a) and 9(b), respectively.

Exploiting the ability to bin Δx and Δy into 0.1-pixel bins [see histograms at top of Figs. 8(b) and 8(c)], the dimensions of the detector image were expanded from the original 512 × 512 pixels to 5120 × 5120 pixels by sub-dividing each pixel into a 10 × 10 grid. This is referred to as the sub-pixelation method. Each element within this 10 × 10 pixel array retained the same intensity value as its corresponding original pixel. Changes in center positions were corrected on a shot-to-shot basis with 0.1-pixel precision of the original 512 × 512 pixel matrix, utilizing the newly formed 5120 × 5120 pixel matrix. The resulting sum detector image of the fully corrected electron beam is depicted in Fig. 9(c). The normalized radial distributions before and after correction are presented in Figs. 9(d) and 9(f) for both unscattered and scattered electrons. It is evident that a more uniform unscattered electron beam is achieved after full correction [blue line in Fig. 9(d)] compared to the intensity-only corrected data (red line) and the uncorrected data (black line). Notably, oscillations in the fully corrected signal [see Fig. 9(d)] are due to aliasing effects arising from the use of 0.1-pixel bin sizes during the sub-pixelation process. Moreover, the shoulder observed for the (220) Brag peak in the uncorrected data is no longer present in the fully corrected dataset [see gray shaded area in Fig. 9(e)]. Such a shot-to-shot correction offers the capability to correct measured scattering data despite intensity and pointing fluctuations in the primary electron beam. This capability has not been attainable thus far in UED instruments utilizing other types of electron detectors mentioned previously. In Sec. IV C, the efficacy of correcting the electron beam intensity jitter is demonstrated.

### C. Time-overlap between optical-pump and electron-probe

**FIG. 10.**
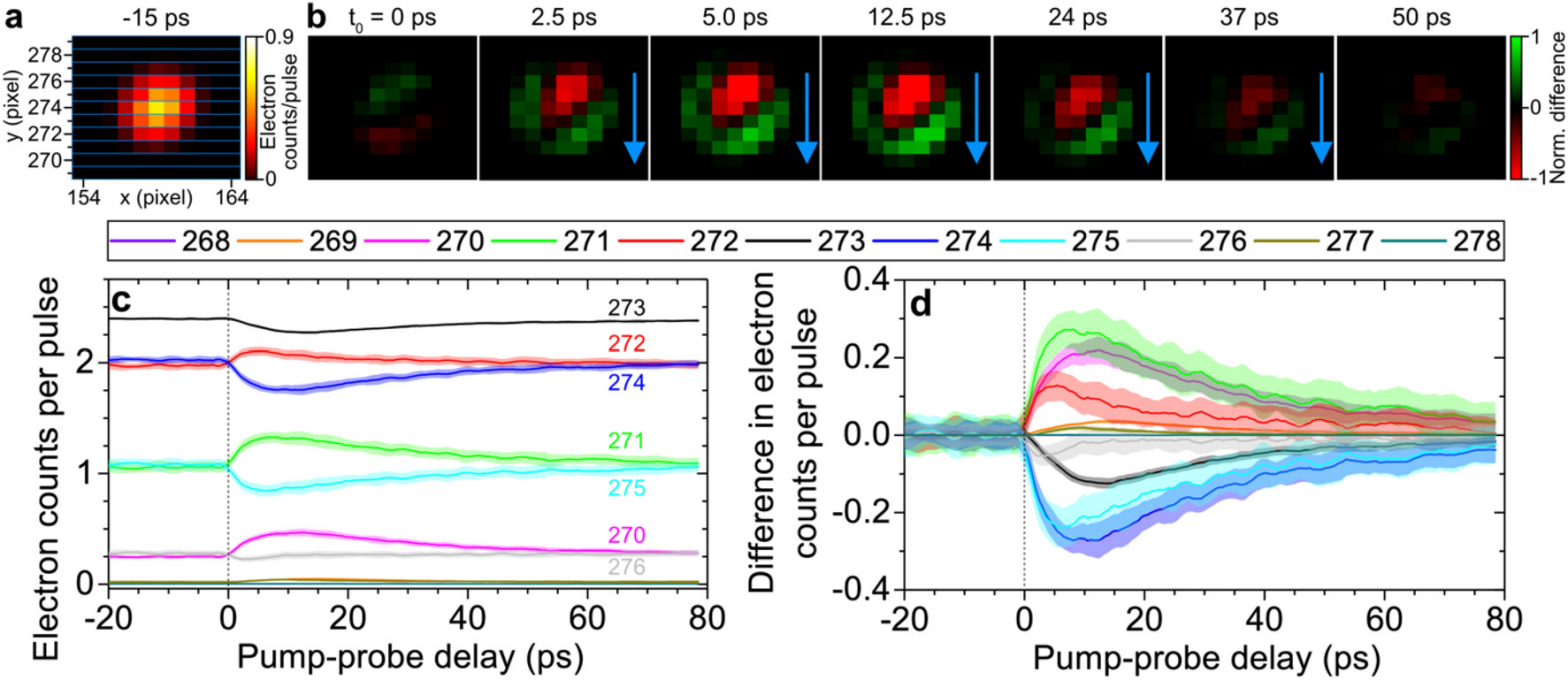
Finding time-zero overlap between the optical-pump and electron-probe pulses by space-charge deflection of the electron beam containing 35 electrons. (a) Detector image of an electron pulse, measured at a pump-probe delay of −15 ps, passing through a copper meshgrid (300 lines/inch) with a 100-μm copper aperture placed in-front of the meshgrid. An optical pump pulse was focused to a ∼100-μm diameter spot on the meshgrid with a fluence of 13.6 mJ/cm^2^. (b) Normalized difference images at positive pump-probe delays, obtained by subtracting the background image at −15 ps from panel (a). Direction of space-charge deflection of electron beam is shown by blue arrows. (c) Electron counts per shot integrated across different horizontal strips of the detector image corresponding to a specific y-pixel integrated over a fixed x-pixel range of 154–164 (see blue shaded rectangles in panel (a) at −15 ps). (d) Same as in panel (c) but with the average counts at negative delays subtracted in each y-pixel horizontal strip.

We investigate the time-resolved pump-probe capability of our UED instrument through space-charge deflection of the primary electron beam (213-fs FWHM simulated, 35 electrons/pulse, 102-μm FWHM diameter at sample) by photoelectrons generated at the surface of a meshgrid (300 lines/inch) using an 800-nm optical pump pulse (60-fs FWHM, 180-μm FWHM, 13.6 mJ/cm^2^).^11^ Figure 10(a) displays the detector image of the electron beam measured at a pump-probe delay of −15 ps. Figure 10(b) presents the difference images of the electron beam measured at positive delays, obtained by subtracting the image at −15 ps each image measured at positive delays. These difference images clearly show that the electron beam is vertically deflected (see blue arrow) due by the photoelectrons generated at the surface of the meshgrid.

To quantify the measured time-resolved signal, we integrate the electron counts in each row of y-pixels across all x-pixels, effectively creating a horizontal strip detector [depicted by blue rectangles in Fig. 10(a) at the delay of −15 ps]. For each pump-probe delay ( Δt), we subtract the pump-probe signal [ $I_{\mathrm{pp}}$; see Fig. 10(b)] by a reference probe-only signal ( $I_{0}$), which provides the difference electron signal [ ΔI; see Fig. 10(c)]. The $I_{0}$ signal is a single image generated from the average of all images measured at negative Δt values. Figures 10(c) and 10(d) reveal a substantial increase in electron counts along the y=270– 272 pixel row strip lines (see green lines). This is accompanied by a decrease in signal along the y=273–276 pixel rows (see light-blue lines) in the first 10-ps after t_0_. These variations in electron signal correspond to the vertical deflection of the electron beam by photoelectrons, slowly returning to its original position by approximately 80 ps.

**FIG. 11.**
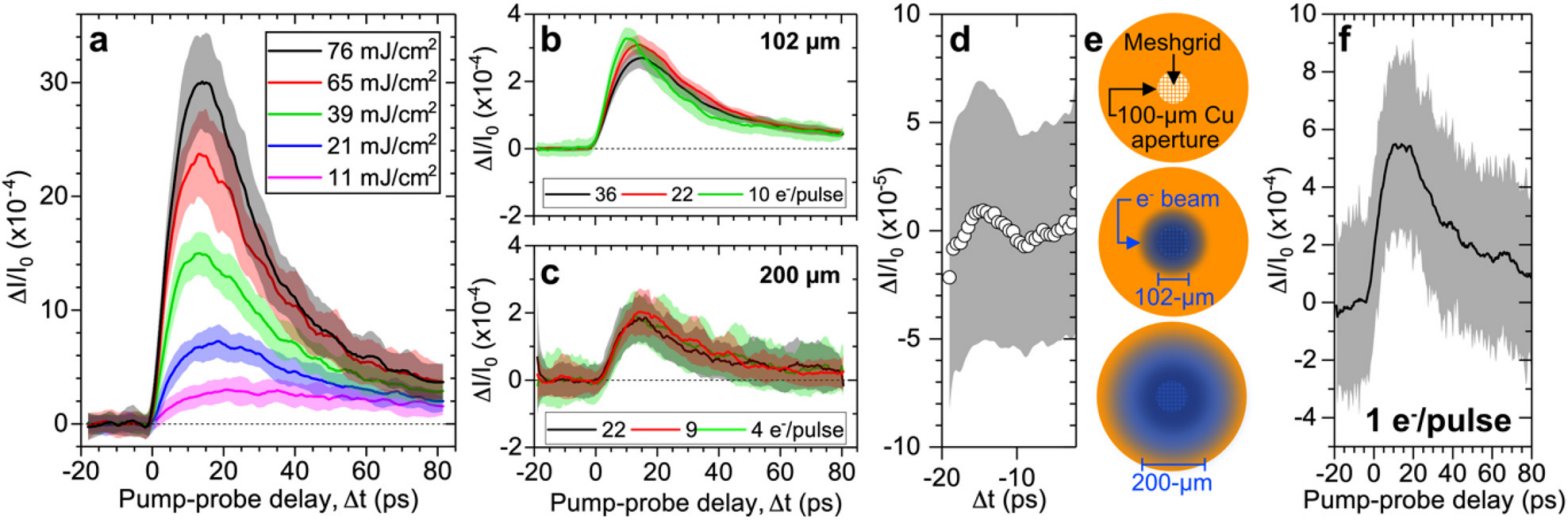
(a) Difference in pump-probe signal relative to the reference probe-only signal, $\Delta I/I_{0}$, as a function of pump-probe delay at different pump fluences. The $\Delta I/I_{0}$ signal was integrated across a circular region of interest with radius of four pixels, and was corrected for fluctuations in electron beam intensity. At all fluences, an electron beam containing 140 electrons had 40 electrons passing through a 100-μm aperture placed in front of the meshgrid sample. $\Delta I/I_{0}$ as a function of pump-probe delay for an electron beam with varying numbers of electrons but with two different FWHM transverse diameters of (b) 102 μm (solid distributions) and (c) 200 μm (dashed distributions) at the sample position. The legend indicates the number of electrons in the beam that passed through the 100-μm aperture. (d) $\Delta I/I_{0}$ at negative pump-probe delays corresponding to the red distribution in panel (b). (e) Schematic of the aperture, meshgrid, and electron beam size. (f) $\Delta I/I_{0}$ as a function of pump-probe delay measured with an electron pulse containing 1 electron before the aperture. The $\Delta I/I_{0}$ signals of panels (b)–(d) and (f) were measured at a constant pump fluence of 13.6 mJ/cm^2^.

Figure 11(a) illustrates the fluence dependence of the time-resolved difference contrast signal, $\Delta I/I_{0}$, arising from space-charge deflection. We define a circular region of interest (ROI) with a radius of four pixels that is centered on the electron beam. On a pixel-by-pixel basis, and at each pump-probe step, we normalize the difference electron signal, ΔI, by the sum of the electron beam to correct for fluctuations in the electron beam intensity. The ΔI signal in each pixel of the ROI is further normalized by $I_{0}$ to derive the difference contrast signal, $\Delta I/I_{0}$. A minimum fluence of 11 mJ/cm^2^ is necessary to detect a discernible $\Delta I/I_{0}$ signal. As the fluence increases, so does the $\Delta I/I_{0}$ signal, reaching up to $3\times 10^{-3}$ (i.e., a 0.3% change relative to the probe-only signal) with a fluence of 76 mJ/cm^2^.

In our investigation, we also study the influence of the electron beam's transverse diameter at the sample position on the measured $\Delta I/I_{0}$ signal. Figures 11(b) and 11(c) show measurements conducted with two different electron beam FWHM diameters: 102 μm [Fig. 11(b)] and 200 μm [Fig. 11(c)]. In both cases, increasing the number of electrons passing through the 100-μm aperture placed in-front of the meshgrid leads to no significant change in the $\Delta I/I_{0}$ signal when considering their corresponding error bars. However, employing a smaller electron beam diameter at the sample [see Fig. 11(e) for a schematic] yields a $\Delta I/I_{0}$ signal with approximately 50% higher contrast. Our results complement previous studies utilizing similar space-charge deflection techniques. Previous investigations often employed electron beams in either shadow imaging mode,^80,81^ characterized by a notably larger beam diameter at the sample, or in an intermediary mode between shadow and reciprocal-space imaging, using a moderately large beam diameter. Notably, our meshgrid space-charge time-resolved signal at 30 kHz only requires a few μJ of pump pulse energy, which is significantly lower than that used in the other methods mentioned above (2 mJ at 1 kHz). Our study demonstrates that achieving a measurable $\Delta I/I_{0}$ signal is equally feasible in reciprocal-space imaging mode. This is accomplished by employing an electron beam with the smallest FWHM diameter, which in our case was equivalent to the aperture's inner diameter. Figure 11(d) shows the $\Delta I/I_{0}$ signal at negative pump-probe delays of the red distribution shown in Fig. 11(b), where the average of the absolute value (standard deviation) of $\Delta I/I_{0}$ is $5\times 10^{-6}$ ( $5\times 10^{-5}$). This represents a 400-fold (40-fold) reduction compared to the minimum $\Delta I/I_{0}$ measurable with EMCCD detection using the MeV-UED instrument at SLAC ( $2\times 10^{-3}$ or 0.2%). The SNR observed in Figs. 11(b) and 11(c) ranges from 60 to 40, which starkly contrasts the SNR of 5 to 2.5 typically obtained with EMCCD detection.^13^ The significant disparity in SNRs emphasizes the critical importance of acquiring electron signals with minimal noise, which is inherently possible with direct electron detection.^82^ For example, the in-pixel digital counting capability of a direct electron detector minimizes inherent sources of noise in the pixel (e.g., gain, integration operations) and readout electronics which have often limited the SNR in charge-integrating analog detectors,^69^ such as CCD and CMOS sensors. Our HiRep-UED instrument employing direct electron detection is primarily limited by shot noise,^64^ arising from the measured signal statistics, as we correct for the source noise,^64^ resulting from fluctuations in the electron beam, by normalizing all intensities measured in every detector image by the sum intensity of the unscattered electron beam.

To further demonstrate the capabilities of our HiRep-UED instrument, we repeated the time-resolved measurements using an electron pulse containing a single electron, measured before the aperture. Figure 11(f) shows similar trends in the time-resolved signal as discussed earlier. Notably, time-resolved signals obtained using 1 electron/pulse (before aperture) are measurable at an average detected count rate of 0.03 electrons/pulse (after aperture). This highlights the capability of direct electron detection to measure time-resolved signals with low SNR at very low count rates.

Additionally, the pulse duration of 1 electron/pulse in our system is simulated to be 174 fs (FWHM) at the sample position. Since the electron pulse is temporally uncompressed, the jitter between the optical pump pulse and electron probe pulse is negligible. Moreover, the velocity mismatch with our 95-keV beam is also negligible for the (<100-nm-thick) solid films of samples studied here. Therefore, the main contributions to the IRF are from the electron probe pulse duration, $t_{e}$ (174-fs), and the optical pump pulse duration, $t_{p}$ (60-fs). Consequently, the lowest IRF of our HiRep-UED instrument is determined to be 184-fs (FWHM) for 1 electron/pulse, as given by

$$\mathrm{IRF}=\sqrt{t_{e}^{2}+t_{p}^{2}}.$$

### D. Time-resolved dynamics in photoexcited aluminum thin film

**FIG. 12.**
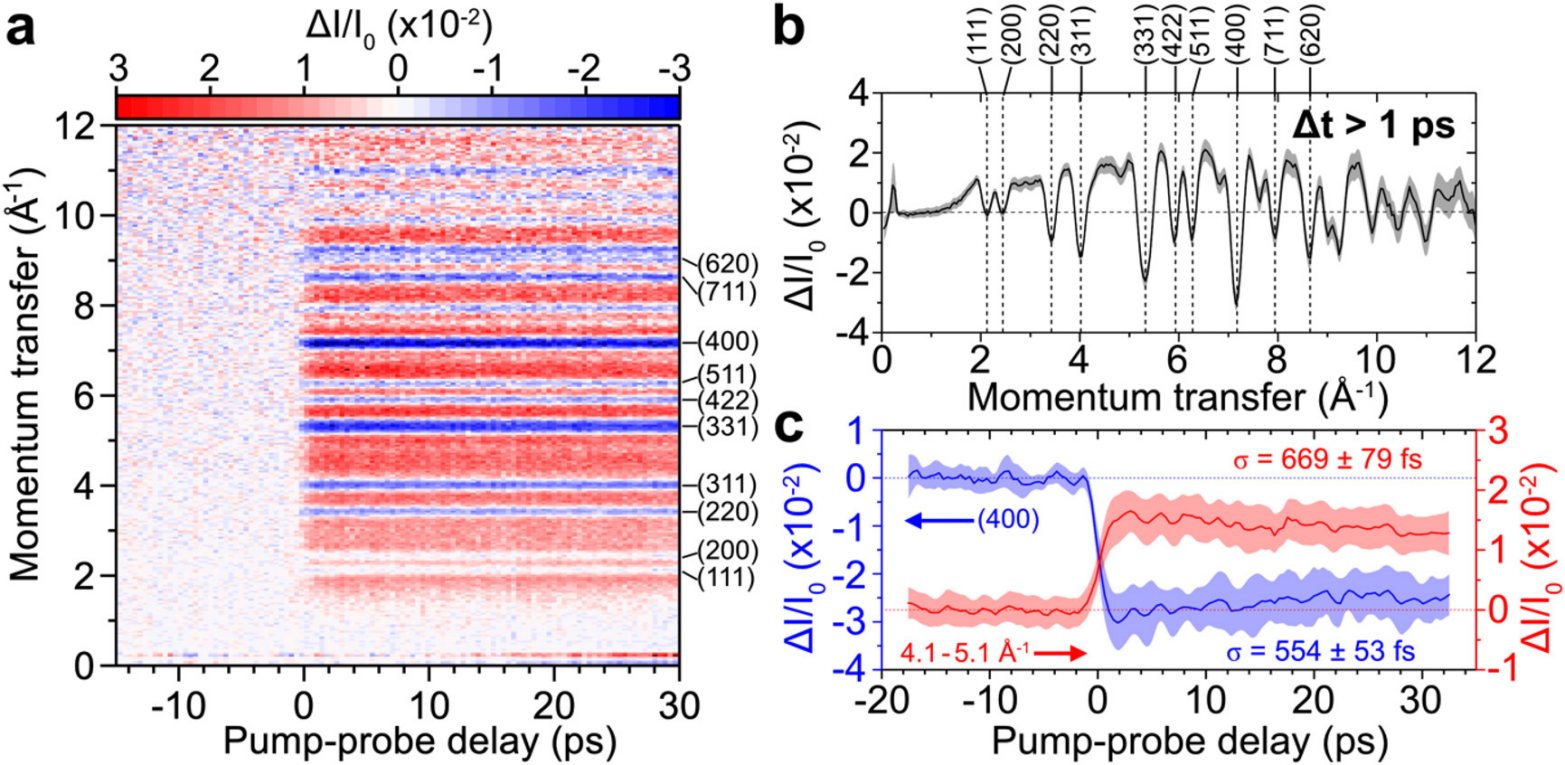
(a) Measured $\Delta I/I_{0}$ signal of 31-nm polycrystalline aluminum thin film optically excited by an 800-nm pump pulse with a fluence of 2 mJ/cm^2^ as a function of momentum transfer and pump-probe delay. Diffraction peaks are labeled. (b) $\Delta I/I_{0}$ as a function of momentum transfer integrated at pump-probe delays of greater than +1 ps. (c) $\Delta I/I_{0}$ as a function of pump-probe delay of the (400) diffraction peak and the $\Delta I/I_{0}$ signal between 4.1 and 5.1 Å^−1^. An electron pulse containing 134 electrons before a 200-μm aperture was used.

We next demonstrate the ability to measure time-resolved elastic electron scattering signals from a 31-nm thin film of polycrystalline aluminum as a prototypical system for expected isotropic gas-phase electron scattering signals. The aluminum sample is optically excited with an 800-nm pulse (<60-fs FWHM, ∼180-μm FWHM diameter, 2 mJ/cm^2^) and probed by a 95-keV electron pulse containing 134 electrons (313-fs FWHM simulated, ∼100-μm FWHM diameter). The expected IRF is 319 fs (FWHM). Figure 12(a) shows the $\Delta I/I_{0}$ signal as a function of momentum transfer and pump-probe delay, while Fig. 12(b) presents the $\Delta I/I_{0}$ signal integrated over pump-probe delays greater than +1 ps.

The first two diffraction peaks of (111) and (200) show no appreciable change in $\Delta I/I_{0}$. While diffraction peaks at higher momentum transfers, from (220) to (620) and higher, show a decrease in $\Delta I/I_{0}$. For example, the $\Delta I/I_{0}$ signal of the (400) diffraction peak decreases by approximately 3% with a time constant of σ=554±53 fs, as shown in Fig. 12(c). This step-function decrease in the $\Delta I/I_{0}$ signal of aluminum is attributed to a transient thermal Debye–Waller effect.^83,84^

Interestingly, electron scattering signals measured at momentum transfers between diffraction peaks, referred to as the diffuse background, show a positive change in $\Delta I/I_{0}$. For example, the integrated $\Delta I/I_{0}$ signal between 4.1 and 5.1 Å^−1^ shows a 1.5% increase in signal with a time constant of σ=669±79 fs, as shown in Fig. 12(c). The diffuse background signal increases simultaneously with the decrease in higher-order diffraction signals. A comparable positive $\Delta I/I_{0}$ signal at positive delays was reported by Siwick *et al.*^33^ during the non-reversible solid-to-liquid phase transition of thin film aluminum at 70 mJ/cm^2^, which required the translation of the sample. In contrast, the data shown in Fig. 12 are composed of multiple pump-probe scans at a much lower fluence of 2 mJ/cm^2^, all measured without translating the sample. At a similar fluence of approximately 2 mJ/cm^2^, previous studies have reported the presence of coherent acoustic phonon modes in thin-film aluminium^83–85^ following photoexcitation by an 800-nm pump pulse. These modes were detected as shifts in the center position of the diffraction peak. In our measurements, however, we do not observe such oscillations in the diffraction peaks.

A comparable increase in the thermal diffuse scattering signal was observed in thin bismuth films by Sokolowski-Tinten *et al.,*^86^ occurring simultaneously with a decrease in the intensity of diffraction peaks. The timescales of the positive and negative changes in signal were also similar. The observed time-resolved changes in bismuth were attributed to a transient Debye–Waller effect, which caused an increase in the atomic displacement after sample excitation, subsequently leading to the diffuse scattering from phonons. Given the similar trend observed in the aluminum data shown in Fig. 12, we attribute the measured time-resolved changes to a similar transient Debye–Waller effect that results in diffuse scattering from phonons. The timescale of these time-resolved changes occurs more than twice faster in aluminum ( σ ∼600 fs) compared to bismuth ( σ ∼1500 fs). This faster timescale can be attributed to stronger electron–phonon coupling in aluminum, which facilitates a more rapid cooling of the hot electrons and a faster equilibration with the lattice in the film.

### E. Simulation of temporal compression in the HiRep-UED instrument

**FIG. 13.**
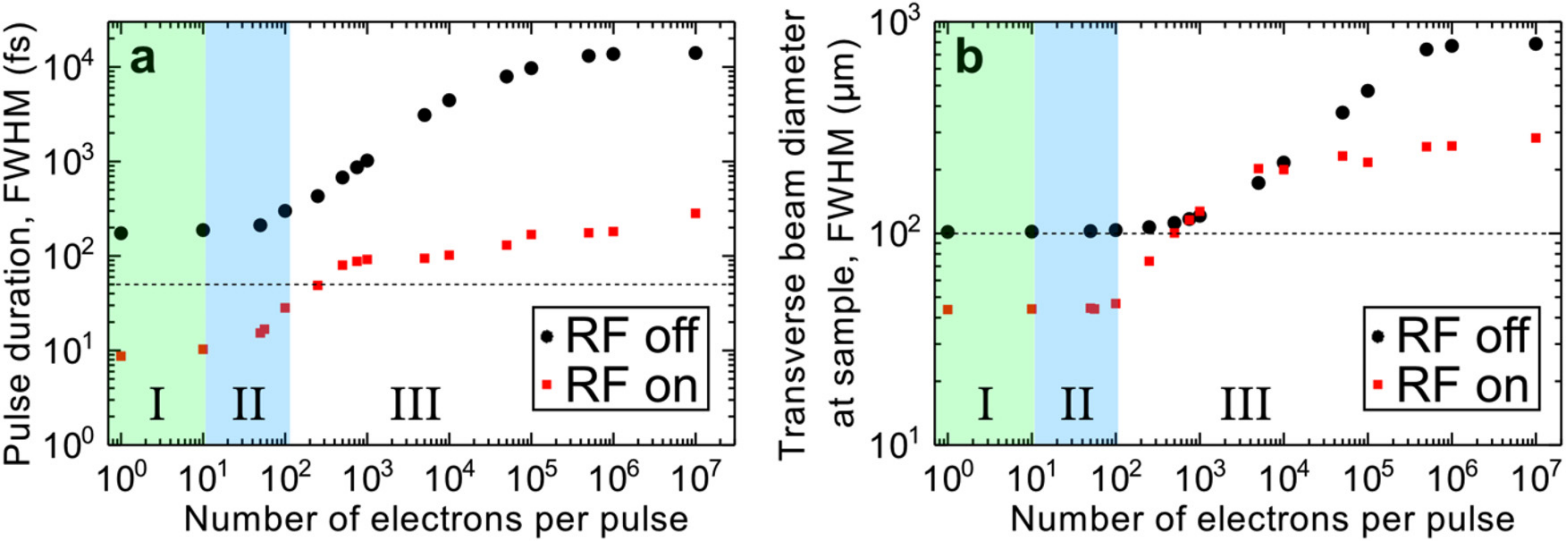
General particle tracer (GPT) simulations of the (a) pulse duration and (b) transverse beam diameter of an electron pulse containing a varied number of electrons at the sample. All values are given in FWHM. Simulated data are shown with the radio frequency (RF) cavity on (red squares) and off (black circles) where the electron beam is temporally compressed and uncompressed, respectively. The UV pulse duration used in the simulations was 60-fs (90-fs) FWHM with the RF cavity on (off). Three regions of space-charge are indicated: (I) no-to-low space-charge (green shaded) (II) low-to-moderate space-charge (blue shaded), and (III) severe space-charge dispersion. Horizontal dashed lines indicate thresholds for an electron beam with a 50-fs duration and a 100-μm transverse diameter at the sample.

In this work so far, we have utilized an electron pulse without temporal compression. However, ongoing efforts are directed toward achieving temporal compression of the electron pulse using an RF compression scheme. While the RF compression of our electron pulse is subject to a future publication, it is still pertinent to explore the anticipated capabilities of our instrument when employing RF-compressed electron beams. Figure 13 shows the FWHM pulse duration and transverse beam diameter at the sample position predicted by GPT simulations for an RF-compressed (red squares) and uncompressed (black circles) electron beam containing from one to 10^7^ electrons.

Here, a UV pulse duration of 60-fs (90-fs) FWHM was used in the simulations with (without) RF compression. The simulations show three distinct regimes of space-charge effects across the single-electron to single-shot operating regimes: no-to-low space-charge (regime I; green shaded), low-to-moderate space-charge (regime II; blue shaded), and severe space-charge (regime III). By implementing RF compression in our instrument, we can achieve pulse durations of 50 fs or less [see dashed line in Fig. 13(a)] with electron beams containing 250 electrons or fewer, as predicted by GPT simulations. This represents a more than tenfold reduction in pulse duration compared to uncompressed electron beams with the same bunch charge. Furthermore, utilizing electron beams containing 250 electrons at 30-kHz results in an electron flux of $7.5\times 10^{6}$ electrons/s, which is more than two times higher than that of the MeV-UED instrument at SLAC operating at 0.36 kHz.^13^ The predicted 50-fs compressed pulse duration would be over two (nearly four) times shorter than that of the MeV-UED instrument at SLAC^13^ (keV-UED setup in Ref. 15). Such a short pulse would enable imaging of nuclear dynamics in gas-phase photochemical reactions that reach completion in less than 500 fs,^87–89^ which have thus far been too rapid to image with existing UED instruments. Moreover, ensuring a minimal transverse diameter of the electron is also crucial to minimize the pump pulse diameter and average power requirements of the laser system operating at high repetition rates. With RF compression, it is anticipated that an electron beam diameter of 100 μm [see dashed line in Fig. 13(b)] could be achieved with a beam containing 500 electrons. In general, GPT simulations predict that electron beams containing 1–10^7^ electrons will exhibit compressed (uncompressed) pulse durations of 9–283 fs (174 fs–14 ps) and transverse diameters of 44–282 μm (102–787 μm). Figure 1 illustrates the range of operating parameters anticipated for our instrument based on GPT simulations (see red dashed lines). These operating regimes are broadly categorized as the ultrashort and high brightness modes. The ultrashort mode is projected to achieve pulse durations of 50 fs or less, while the high brightness mode is capable of pulse durations of below 300 fs but with a substantially higher average current of nearly 70 nA. This average beam current surpasses that of the brightness existing ultrashort keV^44,90^ (MeV^13,17^) electron beam sources by more than one (four) order(s) of magnitude.

## V. CONCLUSIONS

In summary, we introduce a novel high repetition rate UED (HiRep-UED) instrument operating at 30 kHz with direct electron detection. We demonstrate the feasibility of operating in the no-to-moderate space-charge regime at 30 kHz, enabling the acquisition of statistically significant electron signals within a reasonable acquisition time of between a few minutes to up to 90 minutes. Within this regime, the electron beam exhibits relatively low transverse and longitudinal emittance, facilitating the use of temporally uncompressed electron pulses containing 1–140 electrons. This setup allows the measurement of time-resolved signals from photoexcited samples with an instrument response function as low as ∼184 fs (FWHM). Additionally, transverse focusing of the electron beam to a spot diameter as small as ∼100 μm (FHWM) is possible at the sample position. By employing direct electron detection, we demonstrate the ability to measure time-resolved effects in thin film aluminum with a difference contrast $\Delta I/I_{0}$ signal on the 10^−5^ order of magnitude. This high detection sensitivity is made possible by the direct detection of electrons with reduced noise associated with pixel (e.g., gain, integration operations) and readout electronics. Furthermore, direct electron detection enables the measurement of the unscattered primary electron beam, a capability that proves invaluable in correcting for fluctuations in the electron beam's intensity through our experiments under varied experimental conditions.

Our ongoing efforts to improve our setup include the implementation of temporal compression of our electron beam using RF fields generated by a microwave cavity. First, the UV pulse will be chirp-compensated to sub-60-fs using chirped mirrors, suitable for the UV range, possessing negative GDD. Furthermore, the installation of this cavity is complete, and work is underway to optimize its functionality. Additionally, we have implemented an active RF-laser synchronization system based on Ref. 43 to correct fluctuations in the RF-laser timing jitter. The implementation of RF compression into our setup is predicted to extend our capabilities, offering a compressed electron beam with variable duration (9–283 fs) and average current (5 fA to 50 nA). This broad operational range will enable our setup to operate in or between ultrashort and ultrabright modes, catering to diverse experimental requirements. Depending on the timescale of the photo-induced dynamics in gas-phase molecules, the instrument is anticipated to be operated in the ultrashort mode, with an anticipated IRF of less than 100 fs, suitable for studying photochemical reactions completed within 500 fs,^87–89^ or in the ultrabright mode, with up to 10^7^ electrons/pulse, ideal for investigating dynamics on the picosecond or longer timescale, or somewhere between these two modes.

Future iterations of UED setups could benefit from several enhancements. One avenue for improvement involves minimizing the excess transverse momentum spread and thereby reducing the transverse emittance of the electron beam. This would then further reduce the transverse electron beam diameter at the sample position. This could be achieved by adopting new photocathode materials with lower work functions^22,91^ than traditional options like copper^67^ and gold.^34^ Moreover, this would enable the use of visible optical pulse in electron photoemission,^35,91^ allowing to match the photocathode work function,^35^ resulting in the generation of photoelectrons with minimal excess energy and lower emittance. This approach, facilitated by ultrashort visible pulses, represents a highly desired departure from the commonly used ultraviolet pulses. Another important aspect is increasing the acceleration field strength at the photocathode surface to above the typically achieved 10 MV/m in keV-scale UED instruments.^92,93^ While fields exceeding 10 MV/m can produce shorter electron pulses, they also lead to more significant emittance growth at these higher field strengths.^94,95^

Furthermore, our system's current repetition rate of 30 kHz is already well-placed for gas-phase UED measurements planned in the near future. However, scaling the repetition rate of the system to, for example, 100 kHz is feasible using currently available high-average power, femtosecond laser systems. Given that the average power and repetition rate of femtosecond laser systems have continually increased over the last three decades, scaling to hundreds of kHz (or even to the MHz level) is conceivable in the future. Increasing the repetition rate would offer significant benefits, including the ability to utilize electron beams with lower bunch charges. Gas-phase UED measurements could then utilize electron beams experiencing no-to-low space-charge dispersion^10,12,46,50^ and optimal emittance properties but at the hundreds of kHz or 1-MHz repetition rate, particularly when combined with direct electron detection. Additionally, ultrashort electron beams with optimal emittance properties offer promising applications in other areas, such as electron energy loss spectroscopy^23,96^ with the use of streaking fields,^97–99^ dipole magnets,^100,101^ and monochromation techniques.^102^

## Data Availability

The data that support the findings of this study are available from the corresponding author upon reasonable request.
